# Extracorporeal Membrane Oxygenation as a Bridge Therapy Prior to Surgery in a Patient With Echinococcus Cyst Rupture: A Case Report and Literature Review

**DOI:** 10.7759/cureus.61302

**Published:** 2024-05-29

**Authors:** Antonia Kastoris, Christos Efseviou, Dania Rodotheou, Emmanouil Manolis, Marios Tanos

**Affiliations:** 1 Intensive Care Unit, Nicosia General Hospital, Nicosia, CYP; 2 Cardiothoracic Surgery, Nicosia General Hospital, Nicosia, CYP

**Keywords:** veno-venous extracorporeal membrane oxygenation, hydatid cyst, ecmo, echinococcosis, echinococcus cyst

## Abstract

Tapeworms of the genus Echinococcus cause parasitic disease in humans through the ingestion of eggs in contaminated food and water. Rupture of slowly enlarging cysts in the liver, lungs, and other organs can be life-threatening and many deaths are recorded yearly worldwide. Surgery and removal of such cysts remain the most effective treatment. Veno-venous extracorporeal membrane oxygenation (ECMO) routinely placed in the ICU in patients with acute respiratory distress syndrome (ARDS), may provide time and adequate oxygenation for the completion of surgery in echinococcosis cases. In this article, we present a rare case of pulmonary echinococcosis in a young patient requiring ECMO support prior to surgery.

## Introduction

Echinococcosis, a zoonosis, is a parasitic disease caused by tapeworms of the genus Echinococcus and can be classified as cystic (caused by *Echinococcus granulosus*) or alveolar echinococcosis (caused by *Echinococcus multiocularis*). Cystic echinococcosis known as hydatid disease, is caused by infection by a 2-7-mm-long tapeworm. The definitive hosts are carnivorous predators such as dogs, wolves, foxes, and lions that excrete the parasitic eggs from their stools. The intermediate hosts (sheep, cattle, goats, and pigs) are infected by ingesting the eggs that hatch in their digestive system producing planula larvae that are carried via the bloodstream to the liver, lung, brain, and other organs [1].

Human echinococcosis occurs through the ingestion of the parasite eggs in contaminated food and water and the parasite then develops into larval stages in the viscera. Most infections in humans remain asymptomatic but slowly enlarging cysts in the liver, lungs, and other organs may develop that often grow unnoticed and neglected for years. Rupture of the cysts can be life-threatening due to anaphylactic shock. Surgery remains the most effective treatment to remove the cyst and can lead to a complete cure [1]. For cystic echinococcosis, there is an average of 2.2% postoperative death rate for surgical patients, and it was estimated in 2015 to be the cause of death in 19,300 patients, with around 871,000 disability-adjusted life years globally each year [2].

Extracorporeal membrane oxygenation (ECMO) has been used in the past decades in the ICU setting for the management of severe acute respiratory distress syndrome (ARDS), bridging either to recovery or transplantation. It has rarely been used in severe respiratory failure in parasitic worm infections such as pulmonary echinococcosis. The use of ECMO in such infections may prove life-saving and provide sufficient oxygenation during surgery for the removal of Echinococcus cysts. Thoracic surgical intervention without ECMO support may have been impossible to perform due to severe hypoxemia with mechanical ventilation alone [3].

## Case presentation

**Figure 1:**
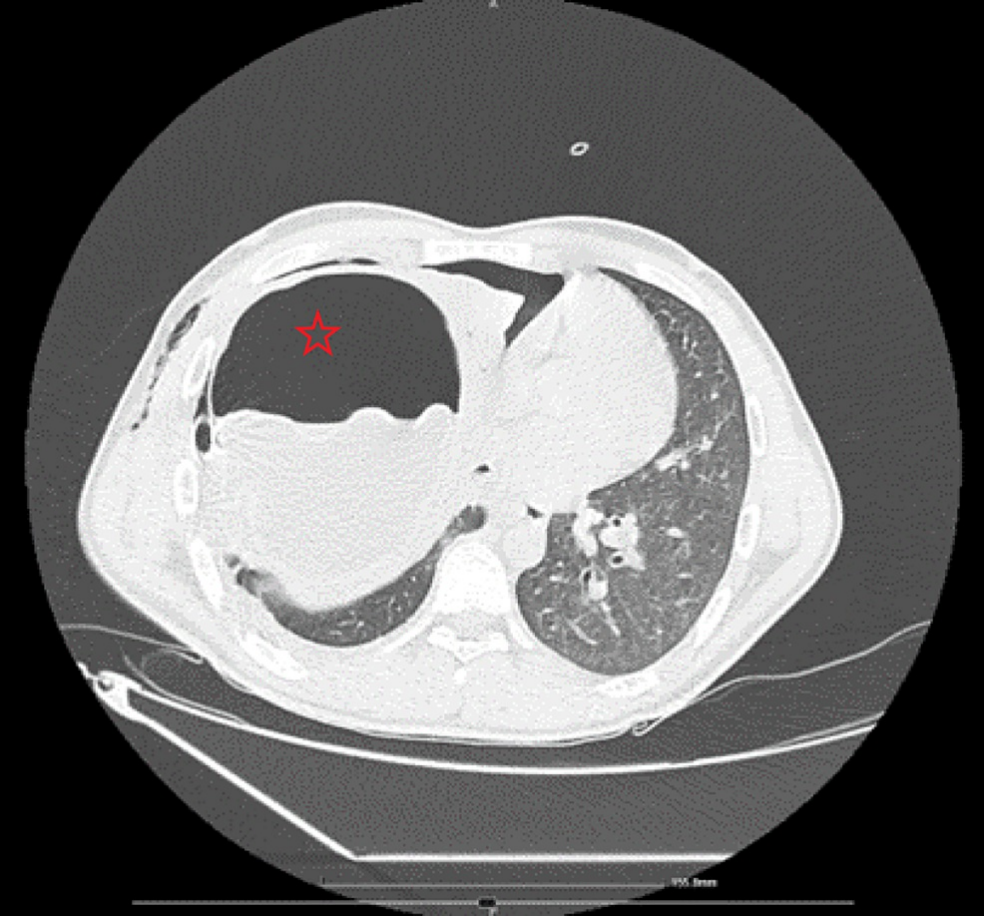
Chest CT of cystic lesion A large fluid-filled cyst and cyst rupture in the right lung.

A 25-year-old male from Syria was admitted to our hospital due to respiratory failure for ECMO consideration. The patient had lived in Cyprus for the past 6 years with an unremarkable medical history, apart from a history of smoking. He also reported weight loss in the past 2 months as well as respiratory symptoms during exercise in the past 5 months. He had been transferred to another hospital the previous day, due to dyspnea, hypoxemia, cough, and right chest pain where on admission a chest tube had been placed in the right hemithorax with improvement of hypoxemia. CT of the thorax revealed a large fluid-filled cystic structure in the right hemithorax (15.5 by 12.5 cm) with cyst rupture (Figure 1).

Due to worsening hypoxemia and respiratory decline, the patient was initially placed on noninvasive ventilation requiring intubation a few hours after admission. The patient continued to exhibit respiratory decline and was subsequently transferred to our hospital.

**Figure 2:**
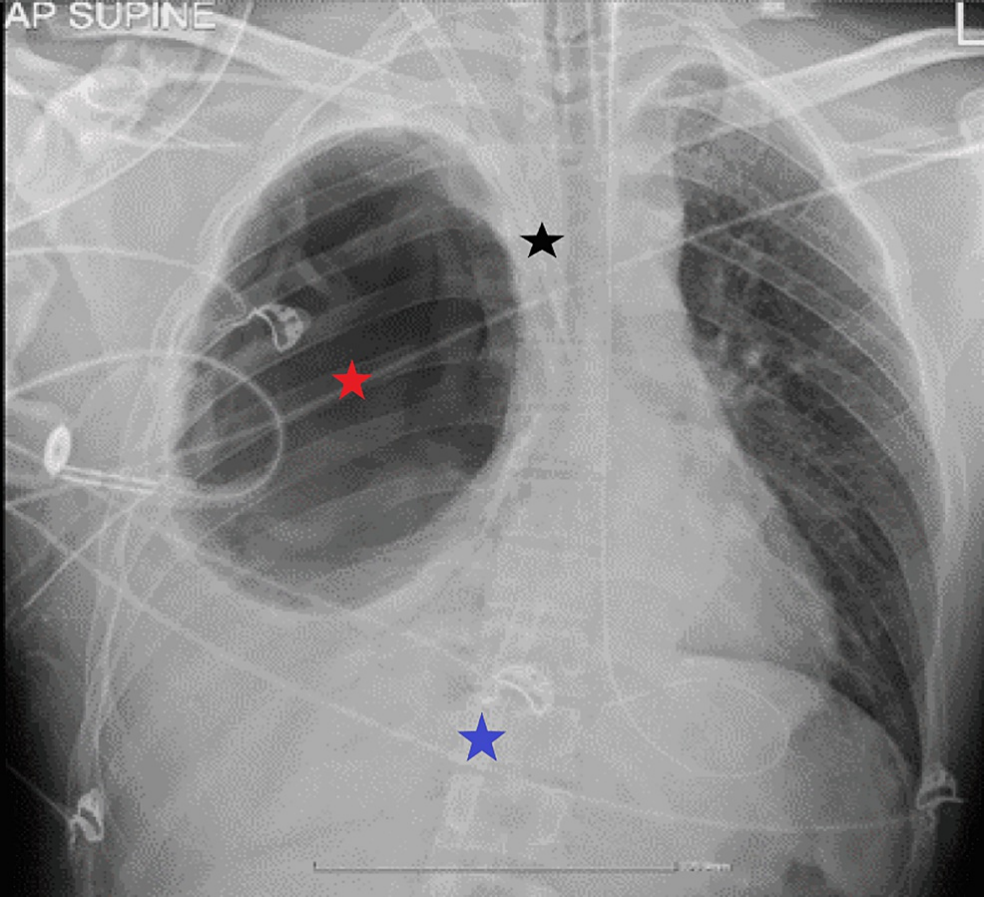
Chest X-ray after ECMO catheter placement Black star: Right subclavian vein ECMO catheter; Blue star: Right femoral vein ECMO catheter; Red star: Echinococcus cyst ECMO: extracorporeal membrane oxygenation

Upon admission to our ICU, the patient continued to present increasing respiratory deterioration and within 3 hours of admission required 100% O_2_ on the ventilator with the lowest SpO_2_ of 77% on the monitor (pre ECMO insertion arterial blood gases PO_2_ 59 mmHg, SaO_2_ 88% with FiO_2_ 100% 6 hours after admission). The patient was on continuous infusion of neuromuscular blocking agents. Prone positioning was not attempted. The decision was made for placement on veno-venous ECMO. With the use of ultrasound guidance a 25 French, 55 cm catheter was placed in the left femoral vein as the drainage cannula, and initially, a 17 French, 15 cm length catheter was placed as a return cannula in the left jugular vein. Due to increasing pressures in the return cannula and high flow on ECMO to maintain oxygenation the return cannula was changed to a 19 French, 15 cm catheter placed in the right jugular vein with ultrasound guidance. In Figure 2, the chest X-ray after catheter placement is shown.

An attempt to change to a double-lumen endotracheal tube resulted in further respiratory decline and was changed back to a single-lumen endotracheal tube. At this time, the patient also exhibited bronchospasm and was placed on ketamine infusion and continuous nebulized adrenaline and salbutamol. The patient had continuous foaming secretions due to cyst rupture. Bronchoscopy revealed increased purulent pulmonary secretions and bronchoalveolar lavage (BAL) was obtained. He required increasing support on ECMO with a maximum of 5.5 L/min to obtain satisfactory PO_2_ levels above 55 mmHg. Table 1 presents inflammation markers on admission and discharge from the ICU. Other laboratory findings were unremarkable.

**Table 1:** Inflammation markers on admission and discharge

Laboratory Test	Laboratory Results	Normal Values
C-reactive protein on admission to the ICU	264 mg/L	below 2 mg/L
White blood cell count on admission to the ICU	11.7 × 10^9^/L	4.5 to 11.0 × 10^9^/L
C-reactive protein on discharge from the ICU	29 mg/L	below 2 mg/L
White blood cell count on discharge from the ICU	9.5 × 10^9^/L	4.5 to 11.0 × 10^9^/L

The patient was initiated with praziquantel 600 mg, three times a day and albendazole 400 mg twice daily as also empiric therapy with meropenem, levofloxacin, and vancomycin awaiting microbiological results from cultures obtained from blood, BAL, bronchial secretions, and urine. The patient required noradrenaline infusion at a rate of 0.67 mcg/kg/min to maintain a mean arterial pressure of 65 mmHg, with continuous tachycardia at a rate of 100-125 beats per minute. Urine output and renal function remained normal.

Surgical cyst extraction was scheduled after ECMO placement. Shortly before surgery, the patient went into cardiac arrest, with pulseless electrical activity, and was successfully resuscitated after 5 minutes and 2 mg of intravenous adrenaline. After resuscitation, the patient was transferred to the operating theater for cyst removal. Placement of a double-lumen endotracheal tube was again unsuccessful, and the patient was ventilated with a conventional endotracheal tube placed in the left main bronchus.

Right-sided thoracotomy was performed, and the cyst was exposed. It was filled with air and sounded like a drum. Opening of the cyst was unavoidable. Compresses with hypertonic 3% saline solution were placed to avoid contamination and distribution of cystic fluid in the thorax. Upon opening the cyst multiple bronchioles were visible. Lobectomy of the right upper lobe was performed due to the extensive damage caused by large bronchopleural fistulas and communication of the ruptured cyst with the rest of the lung as well as the other lung. Two chest tubes were placed in the right hemithorax.

**Figure 3:**
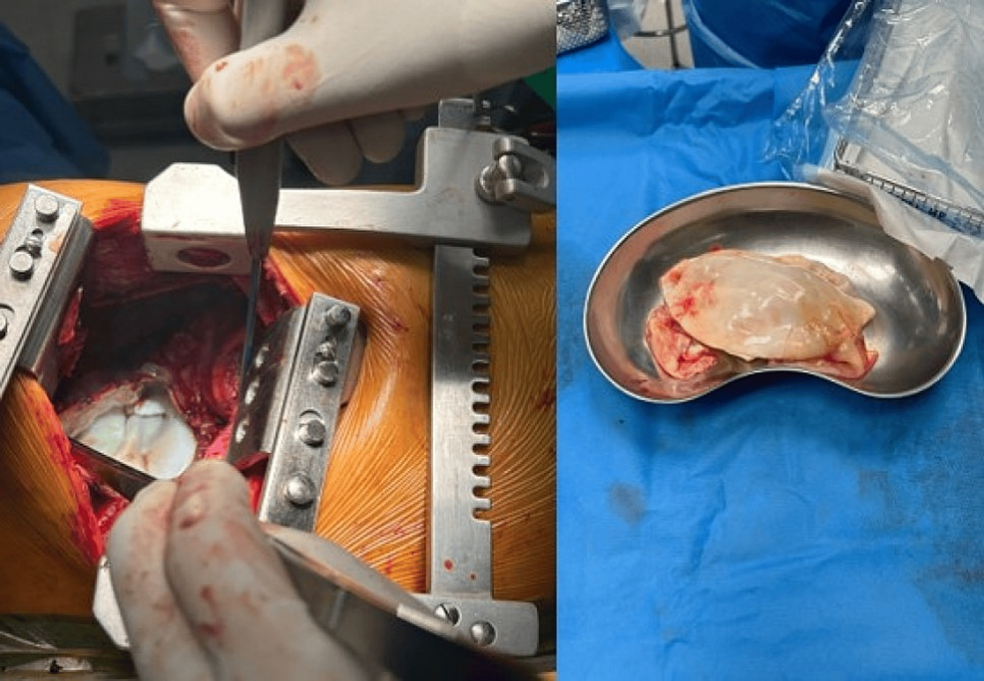
Right-sided thoracotomy and cyst extraction The first photograph on the left shows the cyst after thoracotomy seen as an air-filled white membrane, the right part of the figure shows the full cyst after extraction.

The next day due to continuous respiratory improvement, the patient was extubated while on ECMO with an excellent neurological status and hemodynamically stable. On the 2nd postoperative day, the patient was successfully weaned from the ECMO, and the ECMO cannulas were removed. He was transferred to the thoracic surgery ward on the 4th postoperative day.

Histopathology and microscopy of the fluid and the cyst wall confirmed Echinococcus infection. On confirmation of Echinococcus other antibiotic treatment was stopped.

The postoperative course was uneventful, with no signs of other organ involvement. The patient was discharged home in good condition on the 10th postoperative day with albendazole as sole therapy with follow-up scheduled one month after discharge.

## Discussion

There are very few case reports in the literature regarding the placement of patients with pulmonary echinococcosis on ECMO. A summary of a short review of the literature can be found in Table 2. A search was performed using Google Scholar with the keywords “Echinococcus”, “Echinococcosis”, “hydatid” AND “ECMO” or “Extracorporeal membrane oxygenation”.

**Table 2:** Case reports of patients requiring ECMO due to echinococcosis ICU: intensive care unit; ECMO: extracorporeal membrane oxygenation; ARDS: acute respiratory distress syndrome; DIC: disseminated intravascular coagulation

Author, Date & Place of Publication [Reference]	Age, Sex & Place of Origin of the Patient	ECMO Placement Conditions	Surgical Treatment	Surgery Performed with the Use of ECMO	Outcome
Crnjac et al., 2014, Slovenia [4]	27-year-old, female, Slovenia	ECMO placed 1 day after surgery due to ARDS	Left pneumonectomy	Surgery without ECMO, placed 1st postoperative day	Full recovery
Becker et al., 2017, Germany [5]	23-year-old, female, Bulgaria	Severe hypoxemia	Left lower lung lobectomy	ECMO placed before surgery	Full recover
Dumitrescu et al., 2019, Romania [6]	51-year-old, male, Romania	Inability to complete surgery due to hypoxemia	Removal of cysts in both lungs	ECMO placed during surgery due to hypoxemia	Full recovery
Gomez-Hernandez et al., 2021, Spain [7]	21-year-old, male, Peru	Severe hypoxemia, respiratory acidosis	Right lower lung and left anterior basal segment	ECMO placed one week after ICU discharge	Full recovery
Yousef et al., 2024, Sudan [8]	21-year-old, female, Syrian	Severe hypoxemia	Right middle lobe lobectomy	ECMO placed before surgery	Death on the 8th postoperative day, ARDS, DIC

The use of ECMO in the above-mentioned cases was either as a salvage therapy due to severe hypoxemia or was subsequently left in place for the completion of life-saving thoracic surgery, as it was in our patient. One case used veno-venous ECMO during surgery because of severe hypoxemia, and one case removed ECMO and operated on the patient after ICU discharge due to respiratory failure. Surgical treatment plays a significant role when treating giant hydatid cysts but involves many post and intraoperative complications where severe respiratory failure may be life-threatening. It could be argued that ECMO could be used for even the routine placement of patients requiring thoracic surgical intervention that present preoperatively a high risk for restriction of mechanical ventilation and hypoxemia.

According to the European Centre for Disease Control (ECDC), Cyprus has a low to no number of echinococcosis cases each year in the past years [9]. However, the large migration of the population from other countries has caused a change in infectious diseases found on the island with more cases of HIV, tuberculosis, and parasitosis. Physicians are required thus to be able to effectively treat diseases not common to the island.

ECMO is an ever-evolving tool that despite the great variability worldwide regarding its use and management, has proven to save lives even in dire situations [10]. Its use has expanded to awake patients requiring lung or heart transplantation even before intubation and mechanical ventilation, trauma patients, and even septic patients. Shifts in its possible indications and limitations will become more apparent in the next decades. The use of ECMO in the current case was a ‘bridge to recovery’ through establishing adequate oxygenation during life-saving surgery. It could provide surgeons with possibilities of surgery in patients that before its use could not withstand surgical treatment through mechanical ventilation alone.

## Conclusions

Despite the controversies that may arise with the increasing use of ECMO as a bridge either to recovery or transplantation, its use in infectious diseases other than in severe ARDS such as in our case of echinococcosis could provide a life-saving strategy, especially during necessary surgical procedures.
